# A Practical Treatise on the Management and Diseases of Children

**Published:** 1837-04

**Authors:** 


### Art. IX.

*A Practical Treatise on the Management and Diseases of Children.* By Richard T. Evanson, m.d., Professor of Medicine, and Henry Maunsell, m.d., Professor of Midwifery, in the Royal College of Surgeons in Ireland.—*Dublin,* 1836. 12mo. pp. 257.

The publisher of the volume now before us was certainly justified in informing its authors, "that a concise practical work upon the management and diseases of children was a desideratum in British medical literature;" for, although we possess several works on the subject, we have none calculated to convey to the young practitioner an accurate view of the actual state of our pathological and practical knowledge in this important branch of medical science. The treatise of Drs. Evanson and Maunsell, although (as we shall have occasion to show in the sequel,) not coming entirely up to our idea of what such a work should be, in every respect, is certainly calculated, in a great degree, to supply this defect in our literature. It is, at any rate, the best book we have on the subjects of which it treats; and as, on this account, it is likely to be extensively read by many who are incapable of judging of it critically, we feel it to be our duty to point out freely its defects as well as its merits. In doing so, we trust we shall not be regarded by its very estimable authors as actuated by an unfriendly spirit. This, most assuredly, is not the case; and, in fulfilling our duty towards our readers, we hope we may be able to offer some hints not unworthy the consideration of the authors, when they are called on (as will doubtless be the case, ere long,) to prepare a second edition of their treatise. In executing our task, we shall take a rapid view of the whole range of the subjects treated of; and, if we dwell as long on the parts which we find fault with as on those which we approve, it must be recollected that a systematic treatise, not pretending to originality, does not afford great scope for extract or analysis: we shall notice all that we disapprove of, but we must pass over without comment much that we commend. We may premise, that the work being the combined production of two authors, the different chapters are distinguished in the table of contents by the names of their respective writers.

Chap. i. *On the Peculiarities of the Infant Structure and Constitution;* by Dr. EVANSON. The subject is essentially introductory, and, as such, is handled in a very creditable manner by its author. Much useful general knowledge is brought together in an agreeable and simple style. The physiology of infantile life is well depicted, and appropriately interspersed with practical deductions. We quote the following observations on the cerebro-spinal system as a specimen:

"The general sensibility is acute, and the nervous susceptibility in the infant remarkable; so that all impressions are violently felt, and sympathetic affections are presented in a very aggravated form, and are very prone to occur, constituting a peculiar feature in the infant constitution, which may be emphatically said to be nervous. The large endowment of nervous matter, and its peculiar susceptibility of impression from the softness of its texture, appear to confer this quality, which is never lost sight of in the treatment of infantile disease; a remark as old as the time of Boerhaave, but which is nevertheless too frequently forgotten." (P. 13.)

Chap. ii. *Management and Physical Education of Children;* by Dr. MAUNSELL. In pointing out the necessity for a child enjoying the warmth of its mother's breast, the author has quoted the valuable physiological fact established by Dr. Edwards, "that the heat of mature infants at birth is from three to five degrees less than that of adults, varying between ninety-three and ninety-five degrees; that the heat of premature infants is still less; and that the power of producing heat being in all young animals at its minimum at birth, they have not the same capability of resisting a diminution of their temperature from exposure to cold, as adults." (P. 33.) Dr. Maunsell points out the necessity of removing the *vernix caseosa* from the skin of the new-born child; and he thinks the use of "warm water and a fine sponge" sufficient for the purpose. He does not find that soap is required. In a note he adds, "If any authority were wanting for the removal of vernix caseosa, we have it in the practice which commonly obtains among animals, of licking their offspring immediately after birth." The observation is perfectly correct; but still, as the practice is not *usually* followed in these countries, we are surprised that other means for its removal were not suggested, especially the well-known family recipe of melting a piece of fresh butter in a little thin white wine, as used upon the continent, especially in France, since the time of Portal, (A.D. 1667:) this forms an excellent liniment, and is much better than the cold cream, lard, &c. used in this country. We cannot but defend Dr. Dewees against the charge of using contradictory arguments, merely because he recommends giving the child a teaspoonful or two of molasses and warm water, to assist the evacuation of the meconium, and at the same time observes that the first milk is purgative; because he adds, "*this rarely fails to succeed, especially when aided by the early secretion of the mother's milk.*" Dr. Maunsell, to our surprise, questions this fact of the purgative quality of the first milk, and founds his opinion on that of Professor Joerg, of Leipzig; an authority of but little weight with us, more especially when opposed by such names as C. White, Boer, Dewees, Naegelé and others. We conceive that Dr. Dewees has not rendered himself so open to the charge of recommending "the immediate employment of purgatives," (p. 38,) as the author himself, who says that, "where the abdomen of the child is full, and no evacuation takes place for three or four hours after birth, it will usually be found advantageous to administer half a drachm of oil, provided we can procure it fresh, and free from rancidity."

We feel compelled to dissent entirely with the following passage:

"We have already stated, that the child should be put to the breast as soon as the latter contains anything for it to extract: this generally happens when the mother's system has been relieved, by sleep and ten or twelve hours' rest, from the fever which is always more or less attendant upon labour. Unless there has been *some* secretion of milk, it is better not to apply the child, as it becomes disappointed by continued ineffectual attempts at obtaining nourishment, and there may be subsequently some difficulty in getting it to engage seriously in the business of sucking." (P. 41.)

We ought not to delay applying the child *until* milk has appeared; it should be applied, as Mr. C. White has well expressed it, "in a few hours after delivery, as soon as she has had a little rest, whether there be signs of milk or not." The child thus obtains the first milk, which, as Mr. White observes, is "thin, stimulating, and purgative, for the wise purpose of clearing the child's stomach, bowels, &c." The child draws out the nipple with greater facility, and encourages the earlier and more gradual secretion of milk. The instinctive appetite for sucking is preserved, accumulation of milk in the breast is prevented, and full and permanent contraction of the uterus, which is of such importance, is ensured. Dr. Dewees's observations upon this subject are peculiarly valuable: we would willingly quote them, but our limits do not permit; we therefore refer our readers to his Compendious System of Midwifery, § 504.

Dr. Maunsell's observations on the clothing of children are very judicious, and are made in language so clear and simple as to be well worthy the attention, not only of medical men, but of the general reader.

"We wish we could, as a commentary upon the foregoing passages, adequately depict one of those miserable victims of parental vanity, whose appearance in our streets will sometimes, upon a March or November day, strike cold into our hearts. The cap and feathers set upon, not covering, the child's head, and probably of a colour and richness contrasting mournfully with blue ears, sharpened nose, and shrunken cheeks, in which cold has assumed the features of starvation; the short kilt and Highland hose, exposing between them cracked and quivering knees, altogether require for their description more graphic power than we presume to lay claim to. We hope, however, that we have said enough to call attention to the absurdity of the 'hardening' system, as it is called, and to show that a clothing, regulated so as to obviate the rigors of our climate, is both demanded by our sensations and sanctioned by our knowledge." . . . "In every article of dress, the principle should be carefully followed of placing no constraint upon the motions of any part; for the boy, tight-waisted trousers or braces, and for the girl, stays of all kinds, must be forbidden during the whole period of childhood. The injuries that may be committed upon the organs in the chest and abdomen by the latter article are well known to be of the most serious nature; the chest may be completely altered in shape by a continued pressure, and the lungs diminished in their capacity, while at the same time the stomach and liver are driven from their natural position, and made to press upon the other organs of the abdomen." (P. 59. 60.)

The importance of a well-ventilated room for the child to sleep in, and the necessity for its enjoying a regular and sufficient quantity of rest, are pointed out by the author.

"No definite rules can or ought to be laid down as to the number of hours' sleep to be allowed. One child will require more or less than another; and our only safe guide will be to train it to go to bed shortly after its last meal in the evening, and then to permit it to sleep without disturbance, until it wakes of its own accord upon the following morning." (P. 65.)

We fully agree with the author in reprobating the frequent use, or rather abuse, of calomel in very young infants, but think that the milder preparation of Hydr. cum cretâ ought not here to have passed unnoticed. In most instances where the bowels have been in an unnatural state as respects the *quality* of the evacuations, we think most practitioners must have found small doses of this mercurial, combined with a little soda, (or rhubarb, where there is diarrhœa,) of great service.

We wish that mothers were satisfied with attending to the healthy growth and bodily development of their children, during these first years of their existence; and consider the importance, nay absolute necessity, of pure air, plenty of light, active and cheerful exercise, warm clothing, simple nourishing food, and regular hours; instead of attending so much to the cultivation of the mind at a period of life when the brain is not fully developed, and when its premature and precocious development must inevitably be at the expense of the bodily powers and growth. Dr. Maunsell has very aptly quoted a passage from "The Doctor," a work of great talent, which is strikingly to the point: "We do not, however, attempt to force their intellectual growth. Do not feed them with meat until they have teeth to masticate it. There is a great deal which they ought to learn, can learn, and must learn, before they can or ought to understand it." (P. 76.)

The two succeeding chapters are by Dr. Evanson, viz. Chap. iii. on the *Peculiarities of Disease*, and Chap. iv. on *Infantile Therapeutics.* The former, although containing nothing new, is well digested, and the young practitioner will derive much valuable and practical information from it. Chap. iv. we cannot but consider in great measure unnecessary, and, being of considerable length, has added needlessly, we think, to the size of the book.

Chap. v. *Accidents and Diseases occurring at the Period of Birth, or shortly afterwards;* by Dr. Maunsell. The first article is on *Asphyxia neonatorum.* The subject is hardly so well handled as the reader is entitled to expect, from the excellence of the preceding chapters. The distinction into two species, the *sthenic* and *asthenic,* is totally disregarded; and yet this is of importance. In one there are all the symptoms of plethora and congestion,—the face flushed or purple, features swollen, skin turgid and hot, the cord tense and beating strongly; in the other, the prominent symptoms are those of collapse and atony,—pale face, contracted features, lips blue and flaccid, the jaw fallen, the surface cool, the extremities becoming rapidly cold, the cord flaccid and beating feebly. These are very opposite conditions, and require also very different treatment. In the one case, the loss of a little blood from the cord will seem, as it were, to remove the obstacle, and respiration instantly follows; in the other case, the warm bath and stimulants to the præcordia must be had recourse to.

The subjects of *Imperforate Anus and Vagina* are passed over very cursorily, so as to amount to little more than a mere enumeration of the fact. No allusion is made to Richter's admirable observations. We cannot think the author justified in so doing, because "the case belongs to the general practice of surgery," more especially as he is professor of midwifery to the Dublin College of Surgeons.

The article on *Icterus neonatorum* is liable to the same objection. No mention is made of Boer's valuable observations on this subject: those of Dewees and Henke ought also to have been mentioned.

The article on the *Purulent Ophthalmia of Infants* is simple and practical. Most truly do we agree with the author when he says, "We have, in a large majority of instances, traced it to gonorrhœal or leucorrhœal discharge, existing in the mother before parturition." In his treatment the author has not mentioned warm saturnine lotions, a form of collyrium which we have found of great service.

Chap. vi. *Dentition;* by Dr. Evanson. This subject is rather too briefly handled, considering its importance. The name of John Hunter does not once occur; and many other names of more modern authors, which deserve notice, might also have been mentioned with propriety and advantage.

Chap. vii. *Diseases of the Digestive Organs;* by the same. Among the "affections of the mouth" is aphthæ; but the account of it is neither very clear nor practical. Dr. E., in our opinion, has followed the French writers too closely, and, in imitating their attempts at minute and useless distinctions, has forgotten to give us the more important results of his own experience. Many of these modifications of mucous irritation and inflammation occur only under circumstances of the greatest neglect and exhaustion, and are seldom witnessed in this country. The terms "*Stomatitis*," "*Muguet*," &c. are certainly objectionable in an English treatise; nor do we see why the name and views of M. Billard are to be held up, to the *entire* exclusion of Underwood and Dewees, both of whom have given an infinitely more correct and accurate description of aphthæ. Dr. Evanson has mentioned a species of gangrenous ulceration of the mouth, which we are happy to say we have only known from description.

"A particular form of gangrene of the mouth, without any preceding inflammation, occasionally attacks infants, especially such as are very feeble at birth or broken down by disease. An œdematous circumscribed swelling appears on the cheek, with a central point more or less hard, over which occurs a dark red spot. This spot may appear on the inside or outside of the cheek, and the skin over the œdematous part is characterized by an oily appearance. An eschar forms from within outwards on the central point, and the soft parts mortify, often extensively, down to the bone, so that the parietes of the cheeks and gums are destroyed, falling off in shreds, mixed with a dark sanguineous fluid, and accompanied by a very fetid odour. No disease can be more frightful or more formidable than sloughing of the mouth in children: recovery seems impossible when once the disease has set severely in, the child sinking beneath the constitutional disturbance, independently of the local ravages of the disorder; which, however, are often such as to render recovery not to be desired, so frightful is the deformity necessarily entailed. The fever, which may be at first high, soon sinks into low typhus; the bowels become greatly deranged, and diarrhœa often attends at the close." (P. 222-3.)

We cannot agree with our author in recommending opium as "our chief resource" in the vomiting and purging of infants produced by improper food. Besides the peculiar and dangerous effects which it produces at this early age, and which have been so well described by Dr. John Clarke, opium does harm, by masking the symptoms and concealing the real features of the case from the practitioner. Not only must the diet be changed, but such means taken as shall correct the acidity which so frequently prevails, and induce a healthier action in them. The Hydrarg. c. cretâ, with a little carbonate of soda, is here a very useful combination: after one or two doses, two or three grains of pulv. Rhæi may be substituted with advantage; the rhubarb acts as a stomachic, and tends to check the diarrhœa. Where the purging is extremely violent, mild astringent injections will be of service; but these cannot be ventured upon until a healthy state of the secretions has been established.

In speaking of yellow evacuations, the author has not noticed a species of diarrhœa, which we have occasionally met with, where the intensity and brilliancy of the yellow colour is far beyond that produced by healthy bile; the smell is neither feculent nor sour, but peculiarly faint and sickly; the child rapidly grows thin, and sinks into a state of collapse much sooner than might be expected from the extent of the diarrhœa. We have found mercurials here to be of little or no service. Mild doses of dilute nitric acid, with a gentle tonic in some aromatic water, have produced great relief; the evacuations have become green, and this has been followed by the natural bilious colour. The author considers that bright yellow stools are the results of "high irritation or inflammation of the mucous membrane;" and in this respect he is supported by Dr. Campbell, but the cases just alluded to do not appear to arise from this cause. The utility of nitric acid and tonics has not been unnoticed by the author, but it is to be regretted that the cases in which these medicines are indicated had not been more clearly specified. Dr. Evanson's observations on these medicines deserve attention.

"The bitter infusions make excellent vehicles for the alkalies in such cases, and are not employed in the bowel complaints of children so frequently as they deserve; having the power of restoring the functions of the stomach, while they correct the irritable state of the bowels. Of these bitters, the infusion of hop is particularly eligible for its sedative properties, that of gentian and chamomile flowers as a tonic, and the alkaline carbonates may be exhibited in either of these; but the bitter which most decidedly possesses an astringent power is the infusion of simarouba. This is not compatible with the alkaline carbonates; but one of the most useful compounds that can be employed in protracted cases of diarrhœa, is a combination of simarouba, nitric acid, and opium; a prescription which common repute attributes to the late Dr. Baillie, of London: it may be ordered as follows for children:—℞. Infusi Simaroubæ, ℥iss.; Acidi Nitrici dil. gtt. iv.—vj.; Syrupi Caryophyl. ʒiv.; Tinct. Opii, gtt. vj. One or two teaspoonsful of this mixture to be given in some barley-water three or four times a day.—Other mineral acids have been recommended as astringents in cases of diarrhœa, but the nitrous acid decidedly deserves the preference, appearing to possess some power of allaying the irritability of the mucous membrane." (P. 267.)

This chapter is confessedly on a very important subject, and therefore its length may be considered as unavoidable: it contains much valuable and practical matter; but a number of affections are separately treated of, which ought to be considered as so many results of the same cause and its numerous modifications. It appears to us that there has been some unnecessary repetition, and thus the article rendered longer than it otherwise would have been. By viewing these diseases as it were from one point, we think that the subject would have been rendered more clear and simple.

Chap. viii. *Diseases of the Respiratory Organs;* by Dr. Maunsell.—These affections are well and clearly discussed. The treatment is simple and judicious; but why is no reference made to any English author? why are also Dewees and Henke left entirely unnoticed? Dr. M. appears to consider that the only form of croup is inflammatory, and that there does not exist a spasmodic form of this disease. We cannot agree with him in this respect, as we have distinctly seen an affection which we think entitled to this name, arise from gastric and intestinal irritation; the symptoms being aggravated by antiphlogistic treatment, and relieved by alteratives and laxatives. The real nature of the case will be detected by carefully watching the respiration: we shall find that short intervals of natural breathing occur suddenly every now and then. The spasmodic breathing, the dry, hacking, spasmodic cough of children are modifications of the same state, and arise from the same cause. We think the author, in noticing the opinions of Dr. Ley respecting the disease commonly termed "spasm of the glottis," does not do full justice to that physician; but we have treated this subject so fully in a preceding Number, that we cannot enter on it at present.

Chap. ix. on *Eruptive Fevers;* by Dr. Maunsell. The four articles which this chapter contains,—viz. on Measles, Scarlet Fever, Small-pox, and Chicken-pox,—are well written; the subjects are handled in a clear and practical manner, and are rendered still more interesting and valuable by some well-selected quotations from Sydenham: we read the whole chapter with much satisfaction, and recommend our younger medical brethren to peruse it attentively.

Chap. x. *Vaccination;* by Dr. Maunsell. A note informs us that the "matter of this chapter is now republished from a paper, by Dr. Maunsell, in the fourth volume of the Dublin Medical Journal." It gives a very comprehensive view of the history of, and the more important points connected with, vaccination; and looking upon it, *per se*, as an essay on Vaccination, we must consider it as an excellent digest of whatever is valuable upon this subject; but we cannot think it adapted to a work like the present, which ought to be purely practical, and where it is highly desirable to reduce the size, and therefore the price, within the smallest compass.

Chap. xi. on *Constitutional Disease*, is also by Dr. Maunsell. The subject of scrofula is well handled, although, in our opinion, at greater length than is required for a work like this. His observations on the pathology of this disease are well worthy of notice.

> "These marks [of scrofula] are all such as denote a preponderance of the white tissues and fluids of the body over the red, or, in other words, of lymphatic over the arterial and venous systems. Our physiological knowledge leads us to the inference that the strength, and vitality, and capability of resisting disease possessed by animals (at least, warm-blooded animals,) is in a direct ratio with the red tissues and fluids in their bodies over the white; that, in fact, the white tissues have naturally a lower degree of those qualities than the red; and, the more they abound in the system in relation to the others, the less power will there be for struggling against morbid conditions." (P. 465.)

We perfectly coincide with this view of the subject: it is founded on true physiological as well as pathological principles, and has been for some years our guide in the treatment of these diseases. But why are the names of Drs. Graves and Stokes omitted here? The published lectures of the latter gentleman on this subject are very valuable and original, and have been before the public for some years. Dr. Maunsell's treatment of scrofula is judicious, and founded on the above principles. The combination of sarsaparilla and iodine is excellent, but we think he underrates the value of chalybeate medicines. The combination with iodine in the ioduret of iron, which we can recommend as a very useful form, is not noticed.

The subject of Pemphigus is discussed under the whimsical name of "*Burnt holes!*" In our review of Dr. Collins's work, we deprecated the use of these popular expressions, which, to the greater portion of English readers, must be more or less intelligible. In this point of view, "*green scour*," "*gum fleam*," for gum lancet; "*needing*," for tenesmus; "*stupes*," for fomentations, are all objectionable. We are glad to find that the term "*hippo*," for ipecacuanha, has been scarcely, if at all, used in this work; we could have wished that the words "*muguet*," "*diphtherite*," "*exanthem*," had also been excluded. Dr. Maunsell has only noticed a species of gangrenous pemphigus, described by Dr. Stokes, sen., of Dublin; and takes no notice of the works of Willan, Bateman, &c. Dr. Willan's description of Pemphigus infantilis deserves attention; the more so, as a modification of it has been very prevalent in London during the last two or three years.

Chap. xii. on *Diseases of the Cerebral System*," by Dr. Evanson.—These diseases, we think, might have been better incorporated into the sixth and seventh chapters, with the subjects of which they are so closely connected. Dr. E.'s observations on Hydrocephalus are well arranged and digested, and afford much useful practical matter.

We here close our notice of this work. We repeat, that it possesses much merit; and, from being furnished with a number of well-assorted prescriptions, will prove very useful to the young practitioner, to whom we earnestly recommend its perusal.

The book is closely but well printed; remarkably free from typographical errors; and, considering the quantity of matter it contains, is published at a very moderate price.

---

